# Replication of Gout/Urate Concentrations GWAS Susceptibility Loci Associated with Gout in a Han Chinese Population

**DOI:** 10.1038/s41598-017-04127-4

**Published:** 2017-06-22

**Authors:** Zhiqiang Li, Zhaowei Zhou, Xu Hou, Dajiang Lu, Xuan Yuan, Jie Lu, Can Wang, Lin Han, Lingling Cui, Zhen Liu, Jianhua Chen, Xiaoyu Cheng, Keke Zhang, Jue Ji, Zhaotong Jia, Lidan Ma, Ying Xin, Tian Liu, Qing Yu, Wei Ren, Xuefeng Wang, Xinde Li, Qing-Sheng Mi, Yongyong Shi, Changgui Li

**Affiliations:** 1 The Biomedical Sciences Institute of Qingdao University, Qingdao Branch of SJTU Bio-X Institutes & the Affiliated Hospital of Qingdao University, Qingdao, 266003 P.R. China; 2 Shandong Provincial Key Laboratory of Metabolic Disease, the Affiliated Hospital of Qingdao University & the Metabolic Disease Institute of Qingdao University, Qingdao, 266003 P.R. China; 3 Bio-X Institutes, Key Laboratory for the Genetics of Developmental and Neuropsychiatric Disorders (Ministry of Education) and the Collaborative Innovation Center for Brain Science, Shanghai Jiao Tong University, Shanghai, 200030 P.R. China; 4 Shandong Gout Clinical Medical Center, Qingdao, 266003 P.R. China; 5 School of Kinesiology, Shanghai University of Sport, Shanghai, 200438 P.R. China; 6 Henry Ford Immunology Program, Henry Ford Health System, 1 Ford Place, Detroit, MI 48202 USA; 7 Department of Dermatology, Henry Ford Health System, 1 Ford Place, Detroit, MI 48202 USA; 8 Department of Internal Medicine, Henry Ford Health System, 1 Ford Place, Detroit, MI 48202 USA

## Abstract

Gout is a chronic disease resulting from elevated serum urate (SU). Previous genome-wide association studies (GWAS) have identified dozens of susceptibility loci for SU/gout, but few have been conducted for Chinese descent. Here, we try to extensively investigate whether these loci contribute to gout risk in Han Chinese. A total of 2255 variants in linkage disequilibrium (LD) with GWAS identified SU/gout associated variants were analyzed in a Han Chinese cohort of 1255 gout patients and 1848 controls. Cumulative genetic risk score analysis was performed to assess the cumulative effect of multiple “risk” variants on gout incidence. 23 variants (41%) of LD pruned variants set (n = 56) showed nominal association with gout in our sample (p < 0.05). Some of the previously reported gout associated loci (except *ALDH16A1*), including *ABCG2*, *SLC2A9*, *GCKR*, *ALDH2* and *CNIH2*, were replicated. Cumulative genetic risk score analyses showed that the risk of gout increased for individuals with the growing number (≥8) of the risk alleles on gout associated loci. Most of the gout associated loci identified in previous GWAS were confirmed in an independent Chinese cohort, and the SU associated loci also confer susceptibility to gout. These findings provide important information of the genetic association of gout.

## Introduction

Gout is characteristic of acute arthritis, joint deformity and severe pain caused by deposition of monosodium urate crystals in and around synovial tissue. Elevated serum urate (SU) levels are the most important risk factor of gout. Gout is a multifactorial disease dominated by genetic component with heritability of SU estimated to be 73%^1^. In the recent decade, much effort especially genome-wide association studies (GWAS) have attempted to clarify such contribution and identified dozens of susceptibility loci for SU/gout^2–15^. These studies from different populations (European, African Americans, Japanese and Chinese) provided important clues for better understanding the etiology of gout and some evidences of heterogeneity across populations^2–14^. To our knowledge, only two GWAS, which were published very recently, were conducted for clinically defined gout cases only. One was performed in the Han Chinese population^13^, and the other in the Japanese population^14^. Most of other studies for SU levels (and gout) have primarily been conducted in populations of European descent^2–12, 15^. It is of great interest to replicate the candidate loci for European and/or other populations in Han Chinese population. Many genetic studies for SU/gout have been conducted in Han Chinese^16–19^. However, most of them examined only a small minority of loci. In the present study, we try to determine whether the previously identified SU/gout loci affect susceptibility to gout in Chinese using our recent gout GWAS dataset.

## Methods

### Samples, genotyping and variants selection

All samples including 1255 clinically ascertained gout patients and 1848 healthy controls were of Han Chinese males and signed written informed consent, as described in our recent gout GWAS paper (Supplementary Methods)^13^. Clinical characteristics for the samples were shown in Supplementary Table S1. Genotyping was conducted using Affymetrix Axiom Genome-Wide CHB Array. Detailed methods of quality control and imputation were described as done previously^13^, and a brief description was shown in Supplementary Methods. All the previously identified genome-wide significant loci (p < 5.0 × 10^−8^) related to gout/SU were obtained from the NHGRI GWAS catalog (as to May 12, 2015) and further fine-mapping or mutation analysis studies^20–22^ (Supplementary Tables S2 and S3). Considering the linkage disequilibrium (LD) patterns might differ across different ethnicities for the same susceptibility locus, we included all the available variants those are in LD (*r* ^*2*^ > 0.6) with the genome-wide significant variants based on 1000 Genomes Project datasets. A total of 2255 variants for 1255 gout patients and 1848 controls were kept for subsequent analyses.

### Statistical analysis

Association analysis was performed using the logistic regression with 20 principal components as covariates for correcting the potential population stratification (PCA adjustment analysis, Supplementary Methods). In order to approximate the number of independent variants within each region, we pruned the variants based on LD. A total of 56 LD pruned variants were generated using a *r* ^*2*^ threshold of 0.2. The simple Bonferroni correction for multiple comparisons (n = 2255) was applied, thus 2.22 × 10^−5^ (0.05/2255) was set as the statistical significance level. An uncorrected p value of 0.05 was considered as nominal evidence for association. For the variants in the gout associated loci, an evidence of nominal association was treated as a successful replication, considering pervious evidences for the associations between these loci and gout were solid. The association and LD prune analyses were performed using PLINK^23^. The exact binomial test was performed using R package, by comparing the direction of effect sizes of the tested SNPs between our dataset and the previous reports. The p value was generated under the null hypothesis (H0: p = 0.50). Cumulative genetic risk score analysis was conducted by counting risk alleles in an unweighted method for each individual and calculating the effect on gout risk using logistic regression analysis adjusting for the covariates of principal components.

The study protocol was approved by the Ethics Committee of the Affiliated Hospital, Qingdao University. All procedures were conducted in accordance with the Declaration of Helsinki^24^

### Data availability

The results are available upon request by contacting Li CG or Shi YY. Any additional data (beyond those included in the main text and Supplementary Information) that support the findings of this study are also available from the corresponding author upon request.

### Ethics approval

This study was approved by the relevant ethics review board at the Affiliated Hospital of Qingdao University.

## Results

Based on the LD data of the 1000 Genomes Project datasets for different continental populations (Mixed American, East Asian and European), a total of 2255 variants, which are in LD (*r* ^*2*^ > 0.6) with the previous identified genome-wide significant variants for gout/SU, are tested in this study. After LD (*r* ^*2*^ < 0.2) pruning, 56 LD independent variants were generated. Of the 56 LD independent variants, 23 variants (41%) showed association with gout at p < 0.05 in our sample (Supplementary Table S4). And 11 of the significant variants were from the gout associated loci (*GCKR*, *SLC2A9*, *ABCG2*, *CNIH2*, *MYL2*-*CUX2* (*ALDH2*) and *BCAS3*). The strongest association signal was observed in the *ABCG2* locus (rs1481012, p = 8.96 × 10^−11^, OR = 1.890, Table 1), which is consistent with *Köttgen et al*.’s report (rs1481012, p = 2.00 × 10^−32^, OR = 1.730)^11, 21^. The top significant association was observed at rs11722228 (p = 2.40 × 10^−6^, OR = 1.619) for the *SLC2A9* locus (Table 1). These two variants survived Bonferroni correction for multiple testing (p < 2.22 × 10^−5^). The most highly associated SNP at the *GCKR* locus is rs6547692 (p = 2.20 × 10^−4^, OR = 0.696). The *CNIH2* and *MYL2*-*CUX2* (*ALDH2*) loci are two novel gout loci identified in recent Japanese studies^14, 22^. We observed nominal associations at rs801733 (*CNIH2*, p = 0.026, OR = 0.428) and rs11066008 (*MYL2*-*CUX2* (*ALDH2*), p = 2.94 × 10^−3^, OR = 0.666) (Table 1), which are in strong LD with the previously identified gout associated variants (rs4073582 and rs671, *r* ^*2*^ = 0.96 and 0.79, respectively), and the directions of effects for both variants were consistent with the previous reports^14, 22^. The *BCAS3* locus is one of the novel gout loci identified in our previous report^13^. In addition to this locus, our previous study identified another two gout loci at *RFX3* (rs12236871) and *KCNQ1* (rs179785) (Supplementary Table S5)^13^. Taken together, all gout associated loci that reached the genome-wide significance level in the previous GWAS reports were replicated, except *ALDH16A1*.

**Table 1:** Replication of previously reported gout/SU GWAS associations in a cohort of Han Chinese.

CHR	SNP	BP	A1	Freq.	OR	L95	U95	P	Reported gene(s)	Gout or SU
2	rs6547692	27,734,972	A	0.376/0.455	0.696	0.574	0.844	2.20E-04	*GCKR*	Gout, SU
4	rs11722228	9,915,741	T	0.373/0.298	1.619	1.325	1.979	2.40E-06	*SLC2A9*	Gout, SU
4	rs12505410	89,030,841	G	0.187/0.302	0.571	0.454	0.716	1.33E-06	*ABCG2*	Gout, SU
4	rs1481012	89,039,082	G	0.498/0.305	1.890	1.559	2.291	8.96E-11	*ABCG2*	Gout, SU
6	rs68094823	25,795,971	I^a^	0.169/0.205	0.546	0.421	0.707	4.33E-06	*SLC17A1*	SU
11	rs801733	65,934,549	C	0.015/0.021	0.428	0.202	0.905	0.0264	*CNIH2*	Gout
12	rs11066008	112,140,669	G	0.153/0.201	0.666	0.510	0.871	2.94E-03	*MYL2-CUX2* (*ALDH2*)	Gout
17	rs9895661	59,456,589	C	0.398/0.470	0.594	0.483	0.730	6.94E-07	*BCAS3*	Gout

^a^I, insert; CHR, Chromosome; SNP, dbSNP rs number; BP, Position, based on hg19; A1, minor allele for the whole sample; Freq., frequency of A1 for cases/controls; OR, odds ratio, for A1; L95, the lower endpoint of the 95% confidence interval (CI); U95, the upper endpoint of the 95% confidence interval; P, p value. Reported gene(s), The reported gene(s) in the previous GWAS; Gout or SU, indicating whether the locus found to be associated with gout or SU. All the OR (95% CI) and p values reported in this study were based on the PCA adjustment analysis.

The previously identified susceptibility SNPs were usually considered as more important variants, especially the non-synonymous ones should be given priorities. Because these variants are most likely to have functional consequences, and to be involved in the pathology of gout. We, therefore, performed further analysis for the previously reported non-synonymous variants (Supplementary Table S3). Eight of these reported non-synonymous variants were available in our dataset (Table 2), and all the gout-risk and SU-raising alleles were overrepresented in our cases (Exact binomial test p = 7.81 × 10^−3^). Of them, two variants exhibited statistically significant associations (*ABCG2* Q141K (rs2231142), p = 3.83 × 10^−10^ and *SLC17A1* I269T (rs1165196), p = 1.94 × 10^−5^) and three showed nominal significant associations (*SLC17A4* A318T (rs11754288), p = 9.58 × 10^−5^, *GCKR* L446P (rs1260326), p = 2.23 × 10^−4^ and *ALDH2* E504K (rs671), p = 6.80 × 10^−3^). We noticed the rarity of *SLC2A9* V253I (rs16890979) and *ABCG2* Q126X (rs72552713) (with a minor allele frequency of about 1%) in our sample. As the minor allele frequency of *ABCG2* Q126X is about 1%, the effect of *ABCG2* Q141K will hide the effect by Q126X, we thus performed a multivariate logistic regression only for Q126X and Q141K of *ABCG2* (Supplementary Table S6). Comparing to the univariate analysis, the effect for Q126X was increased (the OR was increased from 1.404 to 2.027), which is consistent with result from similar analysis in the Japanese study^14^. However, it remained non-significant (p = 0.1612). These rare variants often required a larger sample size for detecting significant associations. Similarly, *SLC22A12* G65W (rs12800450) and *ALDH16A1* P476A (rs150414818) were absent in our dataset. Both were low-frequency variants identified to be associated with gout in European and/or Americans samples^9, 10^, however, they were non-polymorphic in the 1000 Genomes Project datasets. Of noted, the other gout associated SNPs identified in the Japanese study^14^ also showed direction-consistent association and with nominal significance in our dataset (rs4073582, p = 0.0339 and rs3775948, p = 3.09 × 10^−3^).

**Table 2:** Association results for the selected important variants.

CHR	SNP	BP	A1	Freq.	OR	L95	U95	P	Reported gene (aa_change)	Gout or SU
**2**	**rs1260326**	**27,730,940**	**C**	**0.378/0.456**	**0.698**	**0.576**	**0.845**	**2.23E-04**	***GCKR*** **(L446P)**	**Gout, SU**
2	rs2307394	148,716,428	T	0.443/0.457	0.918	0.762	1.104	0.3628	*ORC4* (A78S)	SU
4	rs16890979	9,922,167	T	0.011/0.017	0.542	0.229	1.285	0.1644	*SLC2A9* (V253I)	Gout, SU
**4**	**rs3775948**	**9,995,182**	**G**	**0.322/0.402**	**0.738**	**0.604**	**0.903**	**3.09E-03**	***SLC2A9***	**Gout, SU**
**4**	**rs2231142**	**89,052,323**	**T**	**0.496/0.309**	**1.837**	**1.519**	**2.222**	**3.83E-10**	***ABCG2*** **(Q141K)**	**Gout, SU**
4	rs72552713	89,052,957	A	0.009/0.005	1.404	0.5316	3.707	0.4936	*ABCG2* (Q126X)	Gout, SU
**6**	**rs11754288**	**25,776,949**	**A**	**0.155/0.184**	**0.582**	**0.443**	**0.764**	**9.58E-05**	***SLC17A4*** **(A318T)**	**SU**
**6**	**rs1165196**	**25,813,150**	**G**	**0.168/0.203**	**0.570**	**0.441**	**0.738**	**1.94E-05**	***SLC17A1*** **(I269T)**	**SU**
**11**	**rs4073582**	**66,050,712**	**A**	**0.013/0.020**	**0.431**	**0.198**	**0.938**	**0.0339**	***CNIH2***	**Gout**
**12**	**rs671**	**112,241,766**	**A**	**0.116/0.153**	**0.684**	**0.519**	**0.900**	**6.80E-03**	***ALDH2*** **(E504K)**	**Gout**

CHR, Chromosome; SNP, dbSNP rs number; BP, Position, based on hg19; A1, minor allele for the whole sample; Freq., frequency of A1 for cases/controls; OR, odds ratio, for A1; L95, the lower endpoint of the 95% confidence interval (CI); U95, the upper endpoint of the 95% confidence interval; P, p value. Reported gene(s), The reported gene(s) in the previous GWAS; aa_change, amino acid change; Gout or SU, indicating whether the locus found to be associated with gout or SU; The variants with p < 0.05 were indicated in bold. All the OR (95% CI) and p values reported in this study were based on the PCA adjustment analysis.

**Figure 1:**
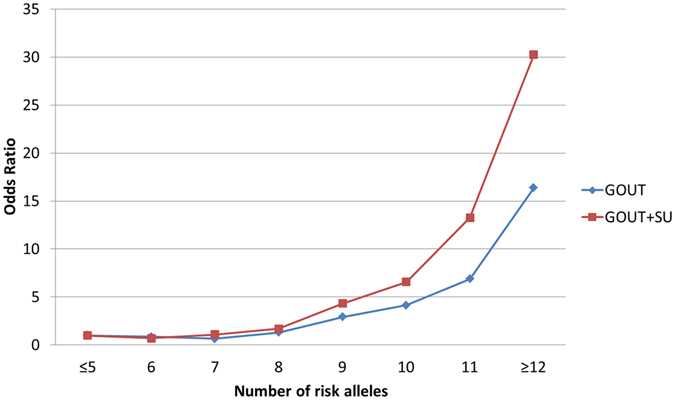
Cumulative effect of the associated variants from gout associated loci and gout + SU associated loci on gout incidence. For the analysis using variants from the gout associated loci (**GOUT**, blue color), seven variants (rs1260326 (L446P)of *GCKR*, rs11722228 of *SLC2A9*, rs12505410 and rs2231142 (Q141K)of *ABCG2*, rs4073582 of *CNIH2*, rs671 (E504K) of *ALDH2* (*MYL2-CUX2*) and rs9895661 of *BCAS3*) were included and eight bins (≤5, 6, 7, 8, 9, 10, 11, and ≥12) were generated. Using the ≤5 bin as the reference category, the OR and 95% CI for each of the other bins (6, 7, 8, 9, 10, 11, and ≥12) were assessed using logistic regression. For the combined analysis of variants from gout and SU associated loci (**GOUT** + **SU**, red color), we also used the ≤5 bin in the gout associated loci analysis as reference, and excluded the individuals with ≤8 risk alleles in the SU associated loci analysis from the test bins (Supplementary Methods).

We then further investigated the cumulative effect for risk alleles of gout associated variants at these loci. Conditional analysis was used to test independent effect for the loci with multiple significant SNPs. The previously identified SNPs (especially the gout associated and non-synonymous ones) were given higher priorities in the analysis for their more important roles. The conditional analysis indicated seven independent variants for the gout associated loci: rs1260326 (L446P) of *GCKR*, rs11722228 of *SLC2A9*, rs12505410 and rs2231142 (Q141K) of *ABCG2*, rs4073582 of *CNIH2*, rs671 (E504K) of *ALDH2* (*MYL2-CUX2*) and rs9895661 of *BCAS3* (Supplementary Table S7), thus we only included these independent variants in the cumulative genetic risk score analysis. We observed a strong increase in the OR with increasing risk allele load (Fig. 1). Comparing to the reference category of having five or fewer risk alleles, ORs for having 8, 9, 10, 11 or 12 more risk alleles were 1.310, 2.925, 4.158, 6.892 and 16.361, respectively (Supplementary Methods and Table S8).

One of the other 12 significant variants from the SU associated loci, rs68094823 (p = 4.33 × 10^−6^, OR = 0.546), was statistically significant after Bonferroni correcting (Table 1). Rs68094823 is an intron variant of *SLC17A1* (also known as *NPT1*), and it’s in strong LD with a previously identified SU associated variant (rs1165151, *r* ^*2*^ = 0.90)^11^. Haplotype analysis suggested that our finding were consistent with previous finding, that is, the gout risk allele is in highly LD with the SU-raising allele. For the *SLC17A1* locus, a common missense variant, rs1165196 (I269T), required special attention. A previous study showed rs1165196 was significantly associated with renal underexcretion gout (a major subtype of gout), but not significant for all gout^20^. In the present study, we provided statistically significant evidence for rs1165196 (p = 1.94 × 10^−5^, OR = 0.570), thus we confirmed the association of rs1165196 with gout (Table 2). The conditional analysis showed that rs1165196 could be the one independent variant in the *SLC17A1* locus (Supplementary Table S7).

For the SU associated loci, we observed 12 independent variants (rs17632159, rs6935612, rs1165196 (*SLC17A1* I269T), rs3734692, rs9321446, rs9314273, rs10821871, rs2361216, rs11172134, rs7978353, rs61168554 and rs11150190). In the cumulative genetic risk score analysis of these variants, we also observed a trend of increase in risk for gout with the growing number of the risk alleles (Supplementary Methods and Figure S1). When setting the reference group as having eight or fewer risk alleles, ORs for the groups having more risk alleles ranged from to 1.644 to 8.884 (Supplementary Table S9). Additionally, we also found an additive effect of the variants from the gout and SU associated loci. The tendency of increasing ORs for cumulative effect of seven variants on gout associated loci escalated, when additional risk alleles on SU associated loci were considered (Fig. 1). Comparing to the reference category as having five or fewer risk alleles at the variants on gout associated loci, ORs ranged from 1.697 to 30.230 for the categories having 8 or more risk alleles on gout associated loci, and at the same time having nine or more risk alleles on SU associated loci (Supplementary Methods and Table S10).

## Discussion

We used a Han Chinese GWAS data of clinically defined gout cases to investigate whether variants associated with gout/SU in other studies can be replicated. For the previously reported gout associated loci, we provided further solid supports that the well-known urate transporter genes (*ABCG2* and *SLC2A9*) and glucokinase regulatory protein gene (*GCKR*) are associated with gout^2, 9, 11, 14, 25^. We, for the first time, replicated the associations of the *CNIH2* and *MYL2*-*CUX2* (*ALDH2*) loci^14, 22^ with gout using a data from a different ethnic group. Moreover, one additional SU associated loci (*SLC17A1*) was found to be associated with gout significantly. The cumulative effects on gout risk for the variants from the gout associated loci were observed in our samples. Similar result was also observed for the variants from the SU associated loci, but the tendency for increasing OR was moderate. Combined analysis of the gout and SU loci presented an additional additive effect.

This study represents a comprehensive evaluation of individual and cumulative effects on risk for gout for previous GWAS identified gout/SU associated loci in a Han Chinese cohort. Replication across different ethnic groups provides stronger evidence for the associations between gout and these loci, and their biological mechanisms will become increasingly important for the understanding of the etiology of gout. However, it should be noted that our data didn’t provide very strong support for most of the loci, which might due to the limited sample size of this study and modest effect sizes of the risk variants. Additional studies with larger sample size and functional studies (mechanism, functional assay and etc.) will be needed to further clarify the roles in gout risk of these loci. Meanwhile, large-scale GWAS of multiple populations are necessary for uncovering the additional genetic factors, especially the ones with small to moderate effect sizes, for further understanding the genetic architecture of gout.

## Electronic supplementary material

Supplementary files
