# Supplementary material for: Replication of Gout/Urate Concentrations GWAS Susceptibility Loci Associated with Gout in a Han Chinese Population
Source: Sci Rep. 2017 Jun 22;7:4094. doi: 10.1038/s41598-017-04127-4 (PMC5481433; doi:10.1038/s41598-017-04127-4)
Supplement: Supplementary file 1 — Supplementary files [file 41598_2017_4127_MOESM1_ESM.doc]

**Replication of Gout/Urate Concentrations GWAS Susceptibility Loci Associated with Gout in a Han Chinese Population**

**Zhiqiang Li,^1,2,3*^ Zhaowei Zhou,^3*^ Xu Hou,^2,4*^ Dajiang Lu,^5*^ Xuan Yuan,^2,4^ Jie Lu,^2,4^ Can Wang,^2,4^ Lin Han,^2,4^ Lingling Cui,^2,4^ Zhen Liu,^2,4^ Jianhua Chen,^3^ Xiaoyu Cheng,^2,4^ Keke Zhang,^2,4^ Jue Ji,^3^ Zhaotong Jia,^2,4^ Lidan Ma,^2,4^ Ying Xin,^2,4^ Tian Liu,^2,4^ Qing Yu,^2,4^ Wei Ren,^2,4^ Xuefeng Wang,^2,4^ Xinde Li,^2,4^ Qing-Sheng Mi,^6,7,8^ Yongyong Shi,^1,2,3#^ Changgui Li^1,2,4#^**

^1^The Biomedical Sciences Institute of Qingdao University, Qingdao Branch of SJTU Bio-X Institutes & the Affiliated Hospital of Qingdao University, Qingdao 266003, P.R. China;

^2^Shandong Provincial Key Laboratory of Metabolic Disease, the Affiliated Hospital of Qingdao University & the Metabolic Disease Institute of Qingdao University, Qingdao 266003, P.R. China;

^3^Bio-X Institutes, Key Laboratory for the Genetics of Developmental and Neuropsychiatric Disorders (Ministry of Education) and the Collaborative Innovation Center for Brain Science, Shanghai Jiao Tong University, Shanghai 200030, P.R. China;

^4^Shandong Gout Clinical Medical Center, Qingdao 266003, P.R. China;

^5^School of Kinesiology, Shanghai University of Sport, Shanghai 200438, P.R. China

^6^Henry Ford Immunology Program, Henry Ford Health System, 1 Ford Place, Detroit, MI, 48202, USA;

^7^Department of Dermatology, Henry Ford Health System, 1 Ford Place, Detroit, MI, 48202, USA;

^8^Department of Internal Medicine, Henry Ford Health System, 1 Ford Place, Detroit, MI, 48202, USA;

*These authors are co-first authors of this study.

^#^Correspondence to: Dr. Changgui Li, Affiliated Hospital of Qingdao University.16 Jiangsu Road, Qingdao 266003, PR China. E-mail: lichanggui@medmail.com.cn; Dr. Yongyong Shi, Bio-X Institutes, Key Laboratory for the Genetics of Developmental and Neuropsychiatric Disorders (Ministry of Education), Shanghai Jiao Tong University, Shanghai 200030, P.R. China. E-mail: shiyongyong@gmail.com.

**SUPPLEMENTARY INFORMATION**

[Supplementary Figure S1. Cumulative effect of the associated variants from SU associated loci on gout incidence 4](#__RefHeading___Toc481677159)

[Supplementary Table S1. Description of samples used in this study 5](#__RefHeading___Toc481677160)

[Supplementary Table S2. All the previously identified genome-wide significant loci (p<5.0×10^−8^) related to gout/SU obtained from the NHGRI GWAS catalog (as to May 12, 2015) 6](#__RefHeading___Toc481677161)

[Supplementary Table S3. Previously identified gout/SU associated functional SNPs 15](#__RefHeading___Toc481677162)

[Supplementary Table S4. Results for the 56 LD independent variants 16](#__RefHeading___Toc481677163)

[Supplementary Table S5. The gout associated SNPs in our previous report 19](#__RefHeading___Toc481677164)

[Supplementary Table S6. Univariate and multivariate logistic regression including Q126X (rs72552713) and Q141K (rs2231142) of *ABCG2* 19](#__RefHeading___Toc481677165)

[Supplementary Table S7. Conditional analysis for independent association in the loci with multiple significant SNPs. 20](#__RefHeading___Toc481677166)

[Supplementary Table S8. Cumulative effect of seven associated variants from the gout associated loci for developing gout 21](#__RefHeading___Toc481677167)

[Supplementary Table S9. Cumulative effect of twelve associated variants from the SU associated loci for developing gout 22](#__RefHeading___Toc481677168)

[Supplementary Table S10. Cumulative effect of seven associated variants from the gout and SU associated loci for developing gout 23](#__RefHeading___Toc481677169)

[Supplementary Methods 24](#__RefHeading___Toc481677170)

[REFERENCES 26](#__RefHeading___Toc481677171)

# Supplementary Figure S1. Cumulative effect of the associated variants from SU associated loci on gout incidence


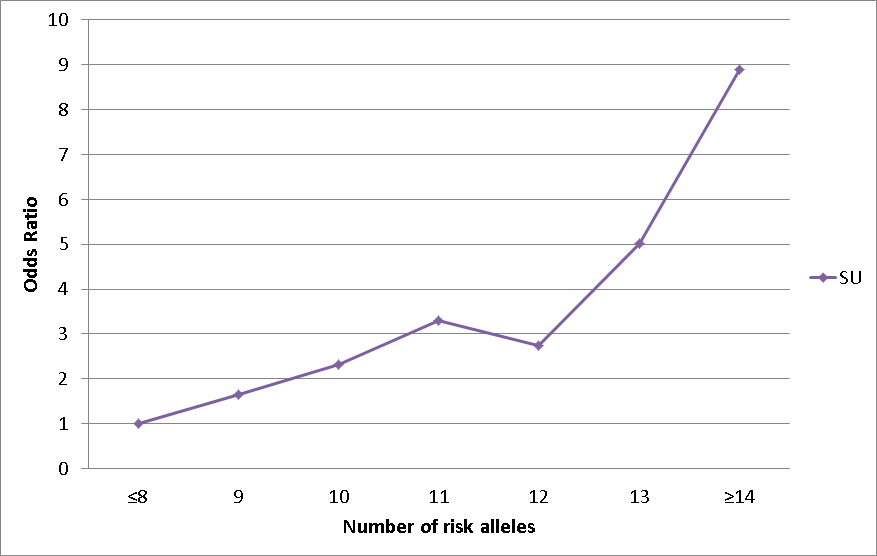


For the analysis using variants from the SU associated loci, 12 variants (rs17632159, rs6935612, rs1165196 (I269T), rs3734692, rs9321446, rs9314273, rs10821871, rs2361216, rs11172134, rs7978353, rs61168554 and rs11150190) were included, and seven bins (≤8, 9, 10, 11, 12, 13 and ≥14) were generated. Using the ≤8 bin as the reference category, the OR and 95% confidence interval (CI) for each of the other bins (9, 10, 11, 12, 13 and ≥14) were assessed using logistic regression.

# Supplementary Table S1. Description of samples used in this study

|  | **N** | **Location** | **Age (years±s.d.)** | **Uric Acid (umol/l±s.d.)** |
| --- | --- | --- | --- | --- |
| Cases | 1255 | Shandong Province | 52.9±13.0 | 458.7±107.6 |
| Controls | 1848 | Northern China^a^ | 53.9±7.92 | - |

^a^Northern China: Shandong, Heilongjiang, Shanxi, Hebei, and Beijing.

# Supplementary Table S2. All the previously identified genome-wide significant loci (p<5.0×10^−8^) related to gout/SU obtained from the NHGRI GWAS catalog (as to May 12, 2015)

| **PUBMEDID** | **First Author** | **Journal** | **Study** | **Disease/Trait** | **Initial Sample Size** | **Replication Sample Size** | **Region** | **CHR** | **BP** | **Reported Gene(s)** | **Strongest SNP-Risk Allele** | **p-Value** | **OR or beta** | **95% CI** |
| --- | --- | --- | --- | --- | --- | --- | --- | --- | --- | --- | --- | --- | --- | --- |
| 19503597 | Kolz M | PLoS Genet | Meta-analysis of 28,141 individuals identifies common variants within five new loci that influence uric acid concentrations. | Uric acid levels | 12,328 European ancestry males, 15,813 European ancestry females | NA | 1q21.1 | 1 | 145709377 | PDZK1 | rs12129861-A | 3.00E-09 | 0.06 | [0.042-0.083] |
| 23263486 | Kottgen A | Nat Genet | Genome-wide association analyses identify 18 new loci associated with serum urate concentrations. | Urate levels | 49,825 European ancestry males, 60,522 European ancestry females | Up to 32,813 European ancestry individuals | 1q21.1 | 1 | 145711327 | PDZK1 | rs1471633-A | 1.00E-29 | 0.059 | [0.049-0.069] |
| 20884846 | Yang Q | Circ Cardiovasc Genet | Multiple genetic loci influence serum urate levels and their relationship with gout and cardiovascular disease risk factors. | Urate levels | 28,283 European ancestry individuals | 22,054 European ancestry individuals | 1q21.1 | 1 | 145711421 | PDZK1 | rs1967017-T | 4.00E-08 | 3.33 | [2.15-4.51] |
| 23263486 | Kottgen A | Nat Genet | Genome-wide association analyses identify 18 new loci associated with serum urate concentrations. | Urate levels | 49,825 European ancestry males, 60,522 European ancestry females | Up to 32,813 European ancestry individuals | 1q22 | 1 | 155179017 | TRIM46, PKLR | rs11264341-T | 6.00E-19 | 0.05 | [0.038-0.062] |
| 25646370 | Matsuo H | Ann Rheum Dis | Genome-wide association study of clinically defined gout identifies multiple risk loci and its association with clinical subtypes. | Gout | 945 Japanese ancestry cases, 1,213 Japanese ancestry controls | 1,048 Japanese ancestry cases, 1,334 Japanese ancestry controls | 2p23.3 | 2 | 27508073 | GCKR | rs1260326-T | 2.00E-12 | 1.36 | [1.25-1.48] |
| 23263486 | Kottgen A | Nat Genet | Genome-wide association analyses identify 18 new loci associated with serum urate concentrations. | Urate levels | 49,825 European ancestry males, 60,522 European ancestry females | Up to 32,813 European ancestry individuals | 2p23.3 | 2 | 27508073 | GCKR | rs1260326-T | 1.00E-44 | 0.074 | [0.063-0.084] |
| 19503597 | Kolz M | PLoS Genet | Meta-analysis of 28,141 individuals identifies common variants within five new loci that influence uric acid concentrations. | Uric acid levels | 12,328 European ancestry males, 15,813 European ancestry females | NA | 2p23.3 | 2 | 27518370 | GCKR | rs780094-T | 1.00E-09 | 0.05 | [0.035-0.068] |
| 20884846 | Yang Q | Circ Cardiovasc Genet | Multiple genetic loci influence serum urate levels and their relationship with gout and cardiovascular disease risk factors. | Urate levels | 28,283 European ancestry individuals | 22,054 European ancestry individuals | 2p23.3 | 2 | 27519736 | GCKR | rs780093-T | 4.00E-17 | 5.15 | [3.95-6.35] |
| 23263486 | Kottgen A | Nat Genet | Genome-wide association analyses identify 18 new loci associated with serum urate concentrations. | Urate levels | 49,825 European ancestry males, 60,522 European ancestry females | Up to 32,813 European ancestry individuals | 2q14.2 | 2 | 120548864 | INHBB | rs17050272-A | 2.00E-10 | 0.035 | [0.023-0.047] |
| 23263486 | Kottgen A | Nat Genet | Genome-wide association analyses identify 18 new loci associated with serum urate concentrations. | Urate levels | 49,825 European ancestry males, 60,522 European ancestry females | Up to 32,813 European ancestry individuals | 2q23.1 | 2 | 147958859 | ACVR2A, ORC4L | rs2307394-T | 2.00E-08 | 0.029 | [0.019-0.039] |
| 20139978 | Kamatani Y | Nat Genet | Genome-wide association study of hematological and biochemical traits in a Japanese population. | Urate levels | 8,868 Japanese ancestry individuals | NA | 2q31.1 | 2 | 169348336 | LRP2 | rs2544390-C | 4.00E-08 | 0.082 | [0.053-0.111] |
| 23263486 | Kottgen A | Nat Genet | Genome-wide association analyses identify 18 new loci associated with serum urate concentrations. | Urate levels | 49,825 European ancestry males, 60,522 European ancestry females | Up to 32,813 European ancestry individuals | 3p21.1 | 3 | 53066198 | MUSTN1, SFMBT1 | rs6770152-T | 3.00E-16 | 0.044 | [0.034-0.054] |
| 20139978 | Kamatani Y | Nat Genet | Genome-wide association study of hematological and biochemical traits in a Japanese population. | Urate levels | 8,868 Japanese ancestry individuals | NA | 4p16.1 | 4 | 9914117 | SLC2A9 | rs11722228-T | 7.00E-24 | 0.164 | [0.13-0.2] |
| 24513273 | Yang B | BMC Med Genomics | A genome-wide association study identifies common variants influencing serum uric acid concentrations in a Chinese population. | Serum uric acid levels | 3,451 Han Chinese ancestry individuals | 8,830 Chinese ancestry individuals | 4p16.1 | 4 | 9914117 | SLC2A9 | rs11722228-T | 9.00E-31 | 0.043 | [0.035-0.051] |
| 18834626 | Dehghan A | Lancet | Association of three genetic loci with uric acid concentration and risk of gout: a genome-wide association study. | Urate levels | 11,847 European ancestry individuals | 11,024 European ancestry individuals, 3,843 African American individuals | 4p16.1 | 4 | 9920543 | SLC2A9 | rs16890979-T | 7.00E-168 | 0.34 | [0.30-0.38] |
| 18759275 | McArdle PF | Arthritis Rheum | Association of a common nonsynonymous variant in GLUT9 with serum uric acid levels in old order amish. | Uric acid levels | 408 Old Order Amish female individuals, 460 Old Order Amish male individuals | Framingham Heart Study (Sample size NR) | 4p16.1 | 4 | 9920543 | GLUT9 | rs16890979-? | 1.00E-11 | 0.44 | [0.32-0.56] |
| 21983786 | Sulem P | Nat Genet | Identification of low-frequency variants associated with gout and serum uric acid levels. | Serum uric acid levels | 15,506 European ancestry individuals | NA | 4p16.1 | 4 | 9921380 | SLC2A9 | rs734553-T | 1.00E-80 | 0.24 | [0.22-0.27] |
| 19503597 | Kolz M | PLoS Genet | Meta-analysis of 28,141 individuals identifies common variants within five new loci that influence uric acid concentrations. | Uric acid levels | 12,328 European ancestry males, 15,813 European ancestry females | NA | 4p16.1 | 4 | 9921380 | SLC2A9 | rs734553-T | 1.00E-41 | 0.22 | [0.188-0.252] |
| 19503597 | Kolz M | PLoS Genet | Meta-analysis of 28,141 individuals identifies common variants within five new loci that influence uric acid concentrations. | Uric acid levels | 12,328 European ancestry males, 15,813 European ancestry females | NA | 4p16.1 | 4 | 9921380 | SLC2A9 | rs734553-T | 1.00E-192 | 0.4 | [0.371-0.423] |
| 24379826 | Voruganti VS | Front Genet | Genome-wide association analysis confirms and extends the association of SLC2A9 with serum uric acid levels to Mexican Americans. | Serum uric acid levels | 632 Mexican American individuals | NA | 4p16.1 | 4 | 9922695 | SLC2A9 | rs6832439-A | 3.00E-08 |  |  |
| 24816252 | Shin SY | Nat Genet | An atlas of genetic influences on human blood metabolites. | Blood metabolite levels | 7,824 European ancestry individuals | NA | 4p16.1 | 4 | 9924068 | SLC2A9 | rs938554-C | 6.00E-93 | 0.035 | [0.031-0.039] |
| 20884846 | Yang Q | Circ Cardiovasc Genet | Multiple genetic loci influence serum urate levels and their relationship with gout and cardiovascular disease risk factors. | Urate levels | 28,283 European ancestry individuals | 22,054 European ancestry individuals | 4p16.1 | 4 | 9925343 | SLC2A9 | rs13129697-T | 4.00E-13 | 1.52 | [1.35-1.69] |
| 20884846 | Yang Q | Circ Cardiovasc Genet | Multiple genetic loci influence serum urate levels and their relationship with gout and cardiovascular disease risk factors. | Urate levels | 28,283 European ancestry individuals | 22,054 European ancestry individuals | 4p16.1 | 4 | 9925343 | SLC2A9 | rs13129697-G | 2.00E-242 | 22.21 | [20.90-23.52] |
| 22229870 | Karns R | Ann Hum Genet | Genome-wide association of serum uric acid concentration: replication of sequence variants in an island population of the Adriatic coast of Croatia. | Uric acid levels | 1,300 European ancestry individuals | NA | 4p16.1 | 4 | 9925343 | WDR1, SLC2A9 | rs13129697-G | 2.00E-19 | 28.99 | [NR] |
| 19260141 | Zemunik T | Croat Med J | Genome-wide association study of biochemical traits in Korcula Island, Croatia. | Biochemical measures | 898 Korculan individuals | NA | 4p16.1 | 4 | 9925343 | SLC2A9 | rs13129697-C | 1.00E-09 | 0.29 | [0.21-0.37] |
| 24816252 | Shin SY | Nat Genet | An atlas of genetic influences on human blood metabolites. | Blood metabolite ratios | Up to 5,591 European ancestry individuals | Up to 1,767 European ancestry individuals | 4p16.1 | 4 | 9925996 | SLC2A9 | rs6838021-A | 6.00E-68 | 0.043 | [0.037-0.049] |
| 18327257 | Vitart V | Nat Genet | SLC2A9 is a newly identified urate transporter influencing serum urate concentration, urate excretion and gout. | Urate levels | 794 European ancestry individuals | 706 European ancestry individuals | 4p16.1 | 4 | 9933120 | SLC2A9 | rs737267-C | 3.00E-09 | 0.88 | [NR] |
| 17997608 | Li S | PLoS Genet | The GLUT9 gene is associated with serum uric acid levels in Sardinia and Chianti cohorts. | Urate levels | 4,305 Sardinian individuals | 1,301 European ancestry individuals | 4p16.1 | 4 | 9934286 | GLUT9 | rs6855911-A | 2.00E-16 | 0.32 | [NR] |
| 23263486 | Kottgen A | Nat Genet | Genome-wide association analyses identify 18 new loci associated with serum urate concentrations. | Gout | 2,115 European ancestry cases, 67,259 European ancestry controls | 1,036 European ancestry cases | 4p16.1 | 4 | 9945032 | SLC2A9 | rs4475146-A | 4.00E-26 | 0.4682 | [0.38-0.55] |
| 21886157 | Suhre K | Nature | Human metabolic individuality in biomedical and pharmaceutical research. | Metabolic traits | 2,820 European ancestry individuals | NA | 4p16.1 | 4 | 9954455 | SLC2A9 | rs4481233-T | 6.00E-34 | 0.074 | [NR] |
| 18179892 | Wallace C | Am J Hum Genet | Genome-wide association study identifies genes for biomarkers of cardiovascular disease: serum urate and dyslipidemia. | Urate levels | 1,955 European ancestry hypertensive individuals | 2,033 European ancestry individuals from 519 families, 1,461 European ancestry twins | 4p16.1 | 4 | 9964756 | WDR1, SLC2A9 | rs7442295-A | 2.00E-15 | 0.02 | [0.02-0.03] |
| 18327256 | Doring A | Nat Genet | SLC2A9 influences uric acid concentrations with pronounced sex-specific effects. | Urate levels | 1,644 European ancestry individuals | 9,947 European ancestry individuals | 4p16.1 | 4 | 9964756 | SLC2A9 | rs7442295-C | 3.00E-70 | 0.35 | [NR] |
| 23703922 | Li WD | Obesity (Silver Spring) | A genome wide association study of plasma uric acid levels in obese cases and never-overweight controls. | Uric acid levels | 487 European ancestry obese individuals, 472 non-obese individuals | NA | 4p16.1 | 4 | 9992591 | SLC2A9 | rs6449213-? | 3.00E-12 |  |  |
| 25646370 | Matsuo H | Ann Rheum Dis | Genome-wide association study of clinically defined gout identifies multiple risk loci and its association with clinical subtypes. | Gout | 945 Japanese ancestry cases, 1,213 Japanese ancestry controls | 1,048 Japanese ancestry cases, 1,334 Japanese ancestry controls | 4p16.1 | 4 | 9993558 | SLC2A9 | rs3775948-G | 6.00E-27 | 1.61 | [1.47-1.75] |
| 21294900 | Charles BA | BMC Med Genomics | A genome-wide association study of serum uric acid in African Americans. | Uric acid levels | 1,017 African American individuals | NA | 4p16.1 | 4 | 9993558 | SLC2A9 | rs3775948-G | 1.00E-08 | 0.211 | [0.14-0.28] |
| 22797727 | Okada Y | Nat Genet | Meta-analysis identifies multiple loci associated with kidney function-related traits in east Asian populations. | Renal function-related traits (urea) | 21,417 East Asian ancestry individuals | 11,657 East Asian ancestry individuals | 4p16.1 | 4 | 9993558 | SLC2A9 | rs3775948-C | 2.00E-65 | 0.1786 | [0.16-0.20] |
| 21943158 | Middelberg RP | BMC Med Genet | Genetic variants in LPL, OASL and TOMM40/APOE-C1-C2-C4 genes are associated with multiple cardiovascular-related traits. | Cardiovascular disease risk factors | 11,683 European ancestry individuals | NA | 4p16.1 | 4 | 10054752 | WDR1 | rs7671266-T | 9.00E-71 | 0.305 | [0.27-0.34] |
| 23703922 | Li WD | Obesity (Silver Spring) | A genome wide association study of plasma uric acid levels in obese cases and never-overweight controls. | Uric acid levels | 487 European ancestry obese individuals, 472 non-obese individuals | NA | 4p16.1 | 4 | 10284677 | SLC2A9 | rs4698014-? | 8.00E-13 |  |  |
| 21943158 | Middelberg RP | BMC Med Genet | Genetic variants in LPL, OASL and TOMM40/APOE-C1-C2-C4 genes are associated with multiple cardiovascular-related traits. | Cardiovascular disease risk factors | 11,683 European ancestry individuals | NA | 4p16.1 | 4 | 10329670 | intergenic | rs4698036-G | 2.00E-52 | 0.257 | [0.22-0.29] |
| 22797727 | Okada Y | Nat Genet | Meta-analysis identifies multiple loci associated with kidney function-related traits in east Asian populations. | Renal function-related traits (urea) | 21,417 East Asian ancestry individuals | 11,657 East Asian ancestry individuals | 4q22.1 | 4 | 88038770 | ABCG2 | rs2725220-C | 4.00E-30 | 0.135 | [0.11-0.16] |
| 25646370 | Matsuo H | Ann Rheum Dis | Genome-wide association study of clinically defined gout identifies multiple risk loci and its association with clinical subtypes. | Gout | 945 Japanese ancestry cases, 1,213 Japanese ancestry controls | 1,048 Japanese ancestry cases, 1,334 Japanese ancestry controls | 4q22.1 | 4 | 88080741 | ABCG2 | rs2728125-C | 7.00E-54 | 2.04 | [1.86-2.23] |
| 23263486 | Kottgen A | Nat Genet | Genome-wide association analyses identify 18 new loci associated with serum urate concentrations. | Gout | 2,115 European ancestry cases, 67,259 European ancestry controls | 1,036 European ancestry cases | 4q22.1 | 4 | 88117930 | ABCG2 | rs1481012-A | 2.00E-32 | 0.5484 | [0.46-0.64] |
| 21983786 | Sulem P | Nat Genet | Identification of low-frequency variants associated with gout and serum uric acid levels. | Gout | 968 European ancestry cases, 40,000 European ancestry controls | NA | 4q22.1 | 4 | 88131171 | ABCG2 | rs2231142-T | 3.00E-12 | 1.67 | [1.43-1.92] |
| 18834626 | Dehghan A | Lancet | Association of three genetic loci with uric acid concentration and risk of gout: a genome-wide association study. | Urate levels | 11,847 European ancestry individuals | 11,024 European ancestry individuals, 3,843 African American individuals | 4q22.1 | 4 | 88131171 | ABCG2 | rs2231142-? | 3.00E-60 | 0.24 | [0.20-0.28] |
| 21983786 | Sulem P | Nat Genet | Identification of low-frequency variants associated with gout and serum uric acid levels. | Serum uric acid levels | 15,506 European ancestry individuals | NA | 4q22.1 | 4 | 88131171 | ABCG2 | rs2231142-T | 2.00E-20 | 0.16 | [0.12-0.19] |
| 23263486 | Kottgen A | Nat Genet | Genome-wide association analyses identify 18 new loci associated with serum urate concentrations. | Urate levels | 49,825 European ancestry males, 60,522 European ancestry females | Up to 32,813 European ancestry individuals | 4q22.1 | 4 | 88131171 | ABCG2 | rs2231142-T | 1.00E-134 | 0.217 | [0.20-0.23] |
| 19503597 | Kolz M | PLoS Genet | Meta-analysis of 28,141 individuals identifies common variants within five new loci that influence uric acid concentrations. | Uric acid levels | 12,328 European ancestry males, 15,813 European ancestry females | NA | 4q22.1 | 4 | 88131171 | ABCG2 | rs2231142-T | 2.00E-18 | 0.22 | [0.171-0.270] |
| 19503597 | Kolz M | PLoS Genet | Meta-analysis of 28,141 individuals identifies common variants within five new loci that influence uric acid concentrations. | Uric acid levels | 12,328 European ancestry males, 15,813 European ancestry females | NA | 4q22.1 | 4 | 88131171 | ABCG2 | rs2231142-T | 1.00E-10 | 0.14 | [0.096-0.181] |
| 24513273 | Yang B | BMC Med Genomics | A genome-wide association study identifies common variants influencing serum uric acid concentrations in a Chinese population. | Serum uric acid levels | 3,451 Han Chinese ancestry individuals | 8,830 Chinese ancestry individuals | 4q22.1 | 4 | 88131171 | ABCG2 | rs2231142-T | 3.00E-42 | 0.045 | [0.037-0.053] |
| 20139978 | Kamatani Y | Nat Genet | Genome-wide association study of hematological and biochemical traits in a Japanese population. | Urate levels | 8,868 Japanese ancestry individuals | NA | 4q22.1 | 4 | 88133515 | ABCG2 | rs4148155-G | 1.00E-13 | 0.121 | [0.09-0.152] |
| 24513273 | Yang B | BMC Med Genomics | A genome-wide association study identifies common variants influencing serum uric acid concentrations in a Chinese population. | Serum uric acid levels | 3,451 Han Chinese ancestry individuals | 8,830 Chinese ancestry individuals | 4q22.1 | 4 | 88139757 | ABCG2 | rs4148152-C | 3.00E-18 | 0.029 | [0.021-0.037] |
| 24513273 | Yang B | BMC Med Genomics | A genome-wide association study identifies common variants influencing serum uric acid concentrations in a Chinese population. | Serum uric acid levels | 3,451 Han Chinese ancestry individuals | 8,830 Chinese ancestry individuals | 4q22.1 | 4 | 88143429 | ABCG2 | rs3114018-T | 4.00E-20 | 0.029 | [0.021-0.037] |
| 23263486 | Kottgen A | Nat Genet | Genome-wide association analyses identify 18 new loci associated with serum urate concentrations. | Urate levels | 49,825 European ancestry males, 60,522 European ancestry females | Up to 32,813 European ancestry individuals | 5q13.2 | 5 | 73135655 | TMEM171 | rs17632159-C | 4.00E-11 | 0.039 | [0.027-0.051] |
| 20884846 | Yang Q | Circ Cardiovasc Genet | Multiple genetic loci influence serum urate levels and their relationship with gout and cardiovascular disease risk factors. | Urate levels | 28,283 European ancestry individuals | 22,054 European ancestry individuals | 6p24.3 | 6 | 7101851 | RREB1 | rs675209-T | 1.00E-09 | 4.39 | [2.98-5.80] |
| 23263486 | Kottgen A | Nat Genet | Genome-wide association analyses identify 18 new loci associated with serum urate concentrations. | Urate levels | 49,825 European ancestry males, 60,522 European ancestry females | Up to 32,813 European ancestry individuals | 6p24.3 | 6 | 7101851 | RREB1 | rs675209-T | 1.00E-23 | 0.0612 | [0.049-0.073] |
| 19503597 | Kolz M | PLoS Genet | Meta-analysis of 28,141 individuals identifies common variants within five new loci that influence uric acid concentrations. | Uric acid levels | 12,328 European ancestry males, 15,813 European ancestry females | NA | 6p22.2 | 6 | 25607343 | SCGN, LRRC16A | rs742132-A | 9.00E-09 | 0.05 | [0.036-0.072] |
| 21943158 | Middelberg RP | BMC Med Genet | Genetic variants in LPL, OASL and TOMM40/APOE-C1-C2-C4 genes are associated with multiple cardiovascular-related traits. | Cardiovascular disease risk factors | 11,683 European ancestry individuals | NA | 6p22.2 | 6 | 25776721 | SLC17A4 | rs11754288-A | 4.00E-09 | 0.084 | [0.057-0.111] |
| 20884846 | Yang Q | Circ Cardiovasc Genet | Multiple genetic loci influence serum urate levels and their relationship with gout and cardiovascular disease risk factors. | Urate levels | 28,283 European ancestry individuals | 22,054 European ancestry individuals | 6p22.2 | 6 | 25812922 | SLC17A1 | rs1165196-G | 5.00E-25 | 6.21 | [5.03-7.39] |
| 23263486 | Kottgen A | Nat Genet | Genome-wide association analyses identify 18 new loci associated with serum urate concentrations. | Urate levels | 49,825 European ancestry males, 60,522 European ancestry females | Up to 32,813 European ancestry individuals | 6p22.2 | 6 | 25821388 | SLC17A1, SLC17A3 | rs1165151-T | 7.00E-70 | 0.091 | [0.081-0.101] |
| 19503597 | Kolz M | PLoS Genet | Meta-analysis of 28,141 individuals identifies common variants within five new loci that influence uric acid concentrations. | Uric acid levels | 12,328 European ancestry males, 15,813 European ancestry females | NA | 6p22.2 | 6 | 25823216 | SLC17A1 | rs1183201-A | 3.00E-14 | 0.06 | [0.459-0.078] |
| 18834626 | Dehghan A | Lancet | Association of three genetic loci with uric acid concentration and risk of gout: a genome-wide association study. | Urate levels | 11,847 European ancestry individuals | 11,024 European ancestry individuals, 3,843 African American individuals | 6p22.2 | 6 | 25870314 | SLC17A3 | rs1165205-? | 4.00E-29 | 0.09 | [0.07-0.11] |
| 23263486 | Kottgen A | Nat Genet | Genome-wide association analyses identify 18 new loci associated with serum urate concentrations. | Urate levels | 49,825 European ancestry males, 60,522 European ancestry females | Up to 32,813 European ancestry individuals | 6p21.1 | 6 | 43836834 | VEGFA | rs729761-T | 8.00E-16 | 0.047 | [0.036-0.058] |
| 21768215 | Tin A | Hum Mol Genet | Genome-wide association study for serum urate concentrations and gout among African Americans identifies genomic risk loci and a novel URAT1 loss-of-function allele. | Urate levels | 8,651 African American individuals | 1,996 African American individuals | 6q23.2 | 6 | 134452416 | intergenic | rs9321453-T | 1.00E-09 | 0.14 | [NR] |
| 23263486 | Kottgen A | Nat Genet | Genome-wide association analyses identify 18 new loci associated with serum urate concentrations. | Urate levels | 49,825 European ancestry males, 60,522 European ancestry females | Up to 32,813 European ancestry individuals | 7q11.23 | 7 | 73442719 | MLXIPL, BAZ1B | rs1178977-A | 1.00E-12 | 0.047 | [0.034-0.06] |
| 23263486 | Kottgen A | Nat Genet | Genome-wide association analyses identify 18 new loci associated with serum urate concentrations. | Urate levels | 49,825 European ancestry males, 60,522 European ancestry females | Up to 32,813 European ancestry individuals | 7q36.1 | 7 | 151708919 | PRKAG2 | rs10480300-T | 4.00E-09 | 0.035 | [0.023-0.047] |
| 23263486 | Kottgen A | Nat Genet | Genome-wide association analyses identify 18 new loci associated with serum urate concentrations. | Urate levels | 49,825 European ancestry males, 60,522 European ancestry females | Up to 32,813 European ancestry individuals | 8p21.2 | 8 | 23919493 | STC1 | rs17786744-A | 1.00E-08 | 0.029 | [0.019-0.039] |
| 23263486 | Kottgen A | Nat Genet | Genome-wide association analyses identify 18 new loci associated with serum urate concentrations. | Urate levels | 49,825 European ancestry males, 60,522 European ancestry females | Up to 32,813 European ancestry individuals | 8q21.11 / 8q21.13 | 8 | 75566533 | HNF4G | rs2941484-T | 4.00E-17 | 0.044 | [0.034-0.054] |
| 23263486 | Kottgen A | Nat Genet | Genome-wide association analyses identify 18 new loci associated with serum urate concentrations. | Urate levels | 49,825 European ancestry males, 60,522 European ancestry females | Up to 32,813 European ancestry individuals | 10q11.23 | 10 | 50886333 | A1CF, ASAH2 | rs10821905-A | 7.00E-17 | 0.057 | [0.043-0.071] |
| 19503597 | Kolz M | PLoS Genet | Meta-analysis of 28,141 individuals identifies common variants within five new loci that influence uric acid concentrations. | Uric acid levels | 12,328 European ancestry males, 15,813 European ancestry females | NA | 10q21.2 | 10 | 59653595 | SLC16A9 | rs12356193-A | 1.00E-08 | 0.08 | [0.051-0.105] |
| 23263486 | Kottgen A | Nat Genet | Genome-wide association analyses identify 18 new loci associated with serum urate concentrations. | Urate levels | 49,825 European ancestry males, 60,522 European ancestry females | Up to 32,813 European ancestry individuals | 10q21.2 | 10 | 59709780 | SLC16A9 | rs1171614-T | 2.00E-28 | 0.079 | [0.065-0.093] |
| 19503597 | Kolz M | PLoS Genet | Meta-analysis of 28,141 individuals identifies common variants within five new loci that influence uric acid concentrations. | Uric acid levels | 12,328 European ancestry males, 15,813 European ancestry females | NA | 11q13.1 | 11 | 64563990 | SLC22A11 | rs17300741-A | 7.00E-14 | 0.06 | [0.046-0.078] |
| 20884846 | Yang Q | Circ Cardiovasc Genet | Multiple genetic loci influence serum urate levels and their relationship with gout and cardiovascular disease risk factors. | Urate levels | 28,283 European ancestry individuals | 22,054 European ancestry individuals | 11q13.1 | 11 | 64566642 | SLC22A11 | rs2078267-C | 2.00E-26 | 6.8 | [5.55-8.05] |
| 23263486 | Kottgen A | Nat Genet | Genome-wide association analyses identify 18 new loci associated with serum urate concentrations. | Urate levels | 49,825 European ancestry males, 60,522 European ancestry females | Up to 32,813 European ancestry individuals | 11q13.1 | 11 | 64566642 | SLC22A11 | rs2078267-T | 9.00E-38 | 0.073 | [0.061-0.085] |
| 19503597 | Kolz M | PLoS Genet | Meta-analysis of 28,141 individuals identifies common variants within five new loci that influence uric acid concentrations. | Uric acid levels | 12,328 European ancestry males, 15,813 European ancestry females | NA | 11q13.1 | 11 | 64589600 | SLC22A12 | rs505802-T | 2.00E-09 | 0.06 | [0.038-0.074] |
| 21768215 | Tin A | Hum Mol Genet | Genome-wide association study for serum urate concentrations and gout among African Americans identifies genomic risk loci and a novel URAT1 loss-of-function allele. | Urate levels | 8,651 African American individuals | 1,996 African American individuals | 11q13.1 | 11 | 64591749 | SLC22A12 | rs12800450-T | 3.00E-16 | 1.19 | [NR] |
| 20139978 | Kamatani Y | Nat Genet | Genome-wide association study of hematological and biochemical traits in a Japanese population. | Urate levels | 8,868 Japanese ancestry individuals | NA | 11q13.1 | 11 | 64673448 | SLC22A12 | rs506338-C | 2.00E-31 | 0.229 | [0.19-0.27] |
| 22797727 | Okada Y | Nat Genet | Meta-analysis identifies multiple loci associated with kidney function-related traits in east Asian populations. | Renal function-related traits (urea) | 21,417 East Asian ancestry individuals | 11,657 East Asian ancestry individuals | 11q13.1 | 11 | 64696613 | SLC22A12 | rs504915-T | 3.00E-63 | 0.2251 | [0.20-0.25] |
| 23263486 | Kottgen A | Nat Genet | Genome-wide association analyses identify 18 new loci associated with serum urate concentrations. | Urate levels | 49,825 European ancestry males, 60,522 European ancestry females | Up to 32,813 European ancestry individuals | 11q13.1 | 11 | 64710591 | NRXN2, SLC22A12 | rs478607-A | 4.00E-11 | 0.047 | [0.033-0.061] |
| 21768215 | Tin A | Hum Mol Genet | Genome-wide association study for serum urate concentrations and gout among African Americans identifies genomic risk loci and a novel URAT1 loss-of-function allele. | Urate levels | 8,651 African American individuals | 1,996 African American individuals | 11q13.1 | 11 | 64778919 | PYGM, RASGRP2, CDC42BPG, NRXN2, MAP4K2, MEN1, SF1 | rs606458-T | 6.00E-11 | 0.18 | [NR] |
| 21768215 | Tin A | Hum Mol Genet | Genome-wide association study for serum urate concentrations and gout among African Americans identifies genomic risk loci and a novel URAT1 loss-of-function allele. | Urate levels | 8,651 African American individuals | 1,996 African American individuals | 11q13.1 | 11 | 64789582 | MAP4K2 | rs493573-A | 2.00E-17 | 0.8 | [NR] |
| 23263486 | Kottgen A | Nat Genet | Genome-wide association analyses identify 18 new loci associated with serum urate concentrations. | Urate levels | 49,825 European ancestry males, 60,522 European ancestry females | Up to 32,813 European ancestry individuals | 11q13.1 | 11 | 65793149 | OVOL1, LTBP3 | rs642803-T | 3.00E-13 | 0.036 | [0.026-0.045] |
| 25646370 | Matsuo H | Ann Rheum Dis | Genome-wide association study of clinically defined gout identifies multiple risk loci and its association with clinical subtypes. | Gout | 945 Japanese ancestry cases, 1,213 Japanese ancestry controls | 1,048 Japanese ancestry cases, 1,334 Japanese ancestry controls | 11q13.2 | 11 | 66283241 | CNIH-2 | rs4073582-G | 6.00E-09 | 1.66 | [1.40-1.96] |
| 23263486 | Kottgen A | Nat Genet | Genome-wide association analyses identify 18 new loci associated with serum urate concentrations. | Urate levels | 49,825 European ancestry males, 60,522 European ancestry females | Up to 32,813 European ancestry individuals | 12q13.13 | 12 | 51857488 | ACVR1B, ACVRL1 | rs7976059-T | 2.00E-09 | 0.032 | [0.022-0.042] |
| 20884846 | Yang Q | Circ Cardiovasc Genet | Multiple genetic loci influence serum urate levels and their relationship with gout and cardiovascular disease risk factors. | Urate levels | 28,283 European ancestry individuals | 22,054 European ancestry individuals | 12q13.3 | 12 | 57415673 | INHBC, R3HDM2 | rs1106766-T | 2.00E-11 | 5.16 | [3.65-6.67] |
| 23263486 | Kottgen A | Nat Genet | Genome-wide association analyses identify 18 new loci associated with serum urate concentrations. | Urate levels | 49,825 European ancestry males, 60,522 European ancestry females | Up to 32,813 European ancestry individuals | 12q13.3 | 12 | 57450266 | INHBC, INHBE | rs3741414-T | 2.00E-25 | 0.072 | [0.058-0.085] |
| 25646370 | Matsuo H | Ann Rheum Dis | Genome-wide association study of clinically defined gout identifies multiple risk loci and its association with clinical subtypes. | Gout | 945 Japanese ancestry cases, 1,213 Japanese ancestry controls | 1,048 Japanese ancestry cases, 1,334 Japanese ancestry controls | 12q24.11 | 12 | 110948323 | MYL2, CUX2 | rs2188380-T | 2.00E-23 | 1.75 | [1.57-1.96] |
| 23263486 | Kottgen A | Nat Genet | Genome-wide association analyses identify 18 new loci associated with serum urate concentrations. | Urate levels | 49,825 European ancestry males, 60,522 European ancestry females | Up to 32,813 European ancestry individuals | 12q24.12 | 12 | 111569952 | ATXN2, PTPN11 | rs653178-T | 7.00E-12 | 0.035 | [0.025-0.045] |
| 23263486 | Kottgen A | Nat Genet | Genome-wide association analyses identify 18 new loci associated with serum urate concentrations. | Urate levels | 49,825 European ancestry males, 60,522 European ancestry females | Up to 32,813 European ancestry individuals | 12q24.31 | 12 | 122141445 | B3GNT4 | rs7953704-A | 3.00E-08 | 0.029 | [0.019-0.039] |
| 23263486 | Kottgen A | Nat Genet | Genome-wide association analyses identify 18 new loci associated with serum urate concentrations. | Urate levels | 49,825 European ancestry males, 60,522 European ancestry females | Up to 32,813 European ancestry individuals | 15q24.2 | 15 | 75866642 | UBE2Q2, NRG4 | rs1394125-A | 3.00E-13 | 0.043 | [0.031-0.055] |
| 23263486 | Kottgen A | Nat Genet | Genome-wide association analyses identify 18 new loci associated with serum urate concentrations. | Urate levels | 49,825 European ancestry males, 60,522 European ancestry females | Up to 32,813 European ancestry individuals | 15q26.3 | 15 | 98727906 | IGF1R | rs6598541-A | 5.00E-15 | 0.043 | [0.031-0.055] |
| 23263486 | Kottgen A | Nat Genet | Genome-wide association analyses identify 18 new loci associated with serum urate concentrations. | Urate levels | 49,825 European ancestry males, 60,522 European ancestry females | Up to 32,813 European ancestry individuals | 16q22.1 | 16 | 69529987 | NFAT5 | rs7193778-T | 8.00E-10 | 0.046 | [0.030-0.062] |
| 22797727 | Okada Y | Nat Genet | Meta-analysis identifies multiple loci associated with kidney function-related traits in east Asian populations. | Renal function-related traits (urea) | 21,417 East Asian ancestry individuals | 11,657 East Asian ancestry individuals | 16q23.2 | 16 | 79612092 | MAF | rs889472-C | 1.00E-09 | 0.0711 | [0.048-0.094] |
| 23263486 | Kottgen A | Nat Genet | Genome-wide association analyses identify 18 new loci associated with serum urate concentrations. | Urate levels | 49,825 European ancestry males, 60,522 European ancestry females | Up to 32,813 European ancestry individuals | 16q23.2 | 16 | 79701090 | MAF | rs7188445-A | 2.00E-09 | 0.032 | [0.022-0.042] |
| 23263486 | Kottgen A | Nat Genet | Genome-wide association analyses identify 18 new loci associated with serum urate concentrations. | Urate levels | 49,825 European ancestry males, 60,522 European ancestry females | Up to 32,813 European ancestry individuals | 17q22 | 17 | 55287427 | HLF | rs7224610-A | 5.00E-17 | 0.042 | [0.032-0.052] |
| 23263486 | Kottgen A | Nat Genet | Genome-wide association analyses identify 18 new loci associated with serum urate concentrations. | Urate levels | 49,825 European ancestry males, 60,522 European ancestry females | Up to 32,813 European ancestry individuals | 17q23.2 | 17 | 61388336 | BCAS3, C17orf82 | rs2079742-T | 1.00E-08 | 0.043 | [0.027-0.059] |
| 21983786 | Sulem P | Nat Genet | Identification of low-frequency variants associated with gout and serum uric acid levels. | Gout | 968 European ancestry cases, 40,000 European ancestry controls | NA |  |  |  | ALDH16A1 | c.1580C>G-G | 2.00E-16 | 3.12 | [2.38-4.17] |
| 20884846 | Yang Q | Circ Cardiovasc Genet | Multiple genetic loci influence serum urate levels and their relationship with gout and cardiovascular disease risk factors. | Urate levels | 28,283 European ancestry individuals | 22,054 European ancestry individuals | 1q23.3 |  |  | ABCG2 | rs2199936-A | 3.00E-23 | 1.86 | [1.64-2.10] |
| 20884846 | Yang Q | Circ Cardiovasc Genet | Multiple genetic loci influence serum urate levels and their relationship with gout and cardiovascular disease risk factors. | Urate levels | 28,283 European ancestry individuals | 22,054 European ancestry individuals | 1q23.3 |  |  | ABCG2 | rs2199936-A | 1.00E-75 | 18.08 | [16.16-20.00] |
| 21983786 | Sulem P | Nat Genet | Identification of low-frequency variants associated with gout and serum uric acid levels. | Serum uric acid levels | 15,506 European ancestry individuals | NA |  |  |  | intergenic | chr1_142697422-C | 5.00E-16 | 0.48 | [0.36-0.60] |
| 21983786 | Sulem P | Nat Genet | Identification of low-frequency variants associated with gout and serum uric acid levels. | Serum uric acid levels | 15,506 European ancestry individuals | NA |  |  |  | ALDH16A1 | c.1580C>G-G | 5.00E-21 | 0.36 | [0.29-0.44] |
| 21943158 | Middelberg RP | BMC Med Genet | Genetic variants in LPL, OASL and TOMM40/APOE-C1-C2-C4 genes are associated with multiple cardiovascular-related traits. | Cardiovascular disease risk factors | 11,683 European ancestry individuals | NA | 1q23.3 |  |  | ABCG2 | rs2199936-A | 2.00E-17 | 0.188 | [0.14-0.23] |

CHR, Chromosome; BP, Position, based on hg19; OR, odds ratio; 95% CI, the 95% confidence interval (CI).

# Supplementary Table S3. Previously identified gout/SU associated functional SNPs

| CHR | SNP | BP | Gene (aa_change) | Reference ID |
| --- | --- | --- | --- | --- |
| 2 | rs1260326 | 27730940 | GCKR (L446P) |  |
| 2 | rs2307394 | 148716428 | ORC4 (A78S) |  |
| 4 | rs16890979 | 9922167 | SLC2A9 (V253I) |  |
| 4 | rs2231142 | 89052323 | ABCG2 (Q141K) |  |
| 4 | rs72552713 | 89052957 | ABCG2 (Q126X) |  |
| 6 | rs11754288 | 25776949 | SLC17A4 (A318T) |  |
| 6 | rs1165196 | 25813150 | SLC17A1 (I269T) |  |
| 11 | rs12800450 | 64359221 | SLC22A12 (G65W) |  |
| 12 | rs671 | 112241766 | ALDH2 (E504K) |  |
| 19 | rs150414818 | 49969006 | ALDH16A1 (P476A) |  |

CHR, Chromosome; BP, Position, based on hg19; aa_change, amino acid change.

# Supplementary Table S4. Results for the 56 LD independent variants

| **CHR** | **SNP** | **BP** | **A1** | **Freq.** | **OR** | **L95** | **U95** | **P** | **Reported gene** | **Gout and/or SU** |
| --- | --- | --- | --- | --- | --- | --- | --- | --- | --- | --- |
| 1 | rs10752826 | 145,602,791 | C | 0.124/0.144 | 0.941 | 0.719 | 1.231 | 0.6565 | PDZK1 | SU |
| 1 | rs900347 | 145,726,727 | G | 0.393/0.390 | 1.076 | 0.889 | 1.303 | 0.4510 | PDZK1 | SU |
| 1 | rs2974929 | 155,197,268 | T | 0.124/0.114 | 1.200 | 0.890 | 1.617 | 0.2312 | TRIM46, PKLR | SU |
| **2** | **rs6547692** | **27,734,972** | **A** | **0.376/0.455** | **0.696** | **0.574** | **0.844** | **2.20E-04** | **GCKR** | **Gout, SU** |
| 2 | rs17050272 | 121,306,440 | A | 0.465/0.487 | 1.038 | 0.863 | 1.248 | 0.6913 | INHBB | SU |
| 2 | rs13021972 | 148,631,232 | A | 0.371/0.395 | 0.879 | 0.719 | 1.074 | 0.2062 | ACVR2A, ORC4 | SU |
| 2 | rs10497025 | 148,662,202 | G | 0.015/0.022 | 0.555 | 0.259 | 1.190 | 0.1304 | ACVR2A, ORC4 | SU |
| 2 | rs2544392 | 170,205,425 | C | 0.456/0.420 | 1.203 | 0.997 | 1.452 | 0.0540 | LRP2 | SU |
| 3 | rs6445559 | 53,099,466 | G | 0.394/0.451 | 0.883 | 0.729 | 1.068 | 0.1986 | MUSTN1, SFMBT1 | SU |
| **4** | **rs11722228** | **9,915,741** | **T** | **0.373/0.298** | **1.619** | **1.325** | **1.979** | **2.40E-06** | **SLC2A9** | **Gout, SU** |
| 4 | rs5028843 | 9,940,806 | A | 0.006/0.016 | 0.330 | 0.101 | 1.076 | 0.0661 | SLC2A9 | Gout, SU |
| **4** | **rs3756231** | **10,025,544** | **G** | **0.319/0.403** | **0.746** | **0.613** | **0.907** | **3.30E-03** | **SLC2A9** | **Gout, SU** |
| **4** | **rs11723742** | **10,111,572** | **G** | **0.081/0.064** | **1.570** | **1.085** | **2.273** | **0.0168** | **SLC2A9** | **Gout, SU** |
| **4** | **rs28441463** | **10,289,577** | **C** | **0.110/0.123** | **0.675** | **0.497** | **0.917** | **0.0120** | **SLC2A9** | **Gout, SU** |
| 4 | rs11736389 | 10,416,360 | C | 0.013/0.017 | 0.568 | 0.252 | 1.280 | 0.1726 | SLC2A9 | Gout, SU |
| 4 | rs2725201 | 88,999,306 | T | 0.456/0.507 | 0.934 | 0.773 | 1.128 | 0.4778 | ABCG2 | Gout, SU |
| 4 | rs79213345 | 89,029,717 | G | 0.151/0.167 | 0.939 | 0.723 | 1.220 | 0.6388 | ABCG2 | Gout, SU |
| **4** | **rs12505410** | **89,030,841** | **G** | **0.187/0.302** | **0.571** | **0.454** | **0.716** | **1.33E-06** | **ABCG2** | **Gout, SU** |
| **4** | **rs1481012** | **89,039,082** | **G** | **0.498/0.305** | **1.890** | **1.559** | **2.291** | **8.96E-11** | **ABCG2** | **Gout** |
| 4 | rs116515496 | 89,041,015 | T | 0.099/0.109 | 1.215 | 0.909 | 1.624 | 0.1884 | ABCG2 | Gout, SU |
| **5** | **rs17632159** | **72,431,482** | **C** | **0.327/0.346** | **0.789** | **0.644** | **0.968** | **0.0227** | **TMEM171** | **SU** |
| 6 | rs488489 | 7,061,116 | A | 0.033/0.040 | 0.806 | 0.490 | 1.326 | 0.3962 | RREB1 | SU |
| 6 | rs113717285 | 7,097,196 | C | 0.047/0.048 | 0.666 | 0.418 | 1.062 | 0.0878 | RREB1 | SU |
| **6** | **rs6935612** | **25,585,844** | **C** | **0.344/0.337** | **1.252** | **1.030** | **1.521** | **0.0238** | **LRRC16A** | **SU** |
| 6 | rs742130 | 25,606,672 | T | 0.063/0.067 | 0.856 | 0.575 | 1.273 | 0.4414 | LRRC16A | SU |
| **6** | **rs68094823** | **25,795,971** | **I** | **0.169/0.205** | **0.546** | **0.421** | **0.707** | **4.33E-06** | **SLC17A1** | **SU** |
| 6 | rs881858 | 43,806,609 | G | 0.208/0.173 | 1.131 | 0.880 | 1.453 | 0.3368 | VEGFA | SU |
| **6** | **rs3734692** | **43,817,791** | **T** | **0.174/0.164** | **1.336** | **1.022** | **1.745** | **0.0338** | **VEGFA** | **SU** |
| **6** | **rs9321446** | **134,760,304** | **C** | **0.127/0.123** | **1.324** | **1.004** | **1.747** | **0.0467** | **intergenic** | **SU** |
| 7 | rs34121855 | 73,040,814 | G | 0.108/0.132 | 0.748 | 0.556 | 1.006 | 0.0546 | MLXIPL, BAZ1B | SU |
| **8** | **rs9314273** | **23,735,559** | **C** | **0.168/0.141** | **1.478** | **1.149** | **1.902** | **2.40E-03** | **STC1** | **SU** |
| 8 | rs2922767 | 76,565,110 | C | 0.420/0.380 | 1.155 | 0.958 | 1.393 | 0.1313 | HNF4G | SU |
| **10** | **rs10821871** | **52,584,110** | **C** | **0.079/0.078** | **1.475** | **1.069** | **2.037** | **0.0181** | **A1CF** | **SU** |
| 11 | rs56369910 | 64,309,574 | T | 0.039/0.033 | 1.173 | 0.703 | 1.959 | 0.5409 | SLC22A11 | SU |
| 11 | rs61884365 | 64,349,466 | A | 0.020/0.023 | 1.410 | 0.780 | 2.549 | 0.2554 | SLC22A11 | SU |
| 11 | rs61884366 | 64,350,528 | A | 0.195/0.224 | 0.813 | 0.634 | 1.044 | 0.1048 | SLC22A11 | SU |
| 11 | rs576076 | 64,360,623 | A | 0.416/0.465 | 0.870 | 0.718 | 1.053 | 0.1513 | SLC22A12 | SU |
| **11** | **rs2361216** | **64,472,319** | **C** | **0.484/0.415** | **1.306** | **1.083** | **1.575** | **5.10E-03** | **NRXN2** | **SU** |
| 11 | rs523200 | 64,532,579 | C | 0.413/0.404 | 0.945 | 0.782 | 1.142 | 0.5561 | SF1 | SU |
| 11 | rs44205 | 65,564,786 | A | 0.160/0.179 | 0.865 | 0.670 | 1.116 | 0.2649 | OVOL1, LTBP3 | SU |
| **11** | **rs801733** | **65,934,549** | **C** | **0.015/0.021** | **0.428** | **0.202** | **0.905** | **0.0264** | **CNIH-2** | **Gout** |
| 12 | rs12227350 | 52,257,466 | T | 0.499/0.491 | 1.186 | 0.977 | 1.439 | 0.0845 | ACVR1B, ACVRL1 | SU |
| **12** | **rs11172134** | **57,645,789** | **A** | **0.112/0.127** | **0.640** | **0.467** | **0.878** | **5.60E-03** | **INHBC, R3HDM2** | **SU** |
| **12** | **rs11065995** | **112,100,755** | **C** | **0.008/0.012** | **0.311** | **0.102** | **0.953** | **0.0410** | **MYL2, CUX2** | **Gout** |
| **12** | **rs11066008** | **112,140,669** | **G** | **0.153/0.201** | **0.666** | **0.510** | **0.871** | **2.94E-03** | **ALDH2** | **Gout** |
| **12** | **rs7978353** | **122,617,989** | **G** | **0.356/0.372** | **1.229** | **1.006** | **1.502** | **0.0435** | **BAZ1B** | **SU** |
| 15 | rs1394125 | 76,158,983 | A | 0.112/0.112 | 1.135 | 0.850 | 1.516 | 0.3898 | UBE2Q2 | SU |
| **15** | **rs61168554** | **99,286,980** | **G** | **0.383/0.351** | **1.219** | **1.004** | **1.480** | **0.0453** | **IGF1R** | **SU** |
| 16 | rs4783580 | 69,552,512 | C | 0.065/0.060 | 1.115 | 0.763 | 1.628 | 0.5737 | NFAT5 | SU |
| 16 | rs9934065 | 79,638,394 | C | 0.484/0.459 | 1.102 | 0.915 | 1.328 | 0.3056 | MAF | SU |
| 16 | rs6564680 | 79,642,269 | C | 0.069/0.082 | 1.082 | 0.764 | 1.531 | 0.6582 | MAF | SU |
| 16 | rs11644836 | 79,712,655 | C | 0.294/0.304 | 1.084 | 0.884 | 1.330 | 0.4377 | MAF | SU |
| 16 | rs12149203 | 79,718,181 | G | 0.063/0.059 | 1.024 | 0.704 | 1.489 | 0.9026 | MAF | SU |
| **16** | **rs11150190** | **79,734,249** | **T** | **0.433/0.445** | **0.808** | **0.666** | **0.981** | **0.0313** | **MAF** | **SU** |
| 17 | rs4793788 | 53,381,796 | C | 0.182/0.163 | 0.956 | 0.748 | 1.221 | 0.7163 | HLF | SU |
| **17** | **rs9895661** | **59,456,589** | **C** | **0.398/0.470** | **0.594** | **0.483** | **0.730** | **6.94E-07** | **BCAS3** | **Gout** |

CHR, Chromosome; SNP, dbSNP rs number; BP, Position, based on hg19; A1, minor allele for the whole sample; Freq., frequency of A1 for cases/controls; OR, odds ratio, for A1; L95, the lower endpoint of 95% CI; U95, the upper endpoint of 95% CI; P, p value. The variants with a p value less than 0.05 were indicated in bold. All the OR (95% CI) and p values reported in this study were based on the PCA adjustment analysis.

# Supplementary Table S5. The gout associated SNPs in our previous report

| **Locus** | **CHR** | **SNP** | **BP** | **A1** | **GWAS** | | **GWAS and REPs** | |
| --- | --- | --- | --- | --- | --- | --- | --- | --- |
|  |  |  |  |  | **P** | **OR** | **P** | **OR** |
| *RFX3* | 9 | rs12236871 | 3,589,117 | G | 4.45 × 10^-5^ | 0.67 | 1.48 × 10^-10^ | 0.81 |
| *KCNQ1* | 11 | rs179785 | 2,781,519 | G | 2.86 × 10^-5^ | 0.66 | 1.28 × 10^-8^ | 0.82 |
| *BCAS3* | 17 | rs11653176^a^ | 59,447,369 | T | 4.01 × 10^-5^ | 0.68 | 1.36 × 10^-13^ | 0.79 |

CHR, Chromosome; SNP, dbSNP rs number; BP, Position, based on hg19; A1, minor allele for the whole sample; OR, odds ratio, for A1; P, p value; ^a^SNP rs11653176 is in LD with rs9895661 (*r^2^*=0.49).

# Supplementary Table S6. Univariate and multivariate logistic regression including Q126X (rs72552713) and Q141K (rs2231142) of *ABCG2*

| **SNP** | **A1** | **Univariate** | | **Multivariate^a^** | |
| --- | --- | --- | --- | --- | --- |
|  |  | **P** | **OR** | **P** | **OR** |
| rs2231142 | T | 3.83E-10 | 1.837 | 5.16E-11 | 1.914 |
| rs72552713 | A | 0.4936 | 1.404 | 0.1612 | 2.027 |

SNP, dbSNP rs number; A1, minor allele for the whole sample; OR, odds ratio, for A1; P, p value. ^a^Logistic regression analysis were performed using a multivariate model including two SNPs.

# Supplementary Table S7. Conditional analysis for independent association in the loci with multiple significant SNPs.

| **CHR** | **SNP** | **BP** | **Test p value** | **Condition on** | **Conditioned** | **Locus** |
| --- | --- | --- | --- | --- | --- | --- |
|  |  |  |  |  | **P value** |  |
| 2 | rs1260326 | 27,730,940 | 2.23E-04 | rs1260326 | NA | *GCKR* |
| 2 | rs6547692 | 27,734,972 | 2.20E-04 |  | 0.3225 |  |
| 4 | rs11722228 | 9,915,741 | 2.40E-06 | rs11722228 | NA | *SLC2A9* |
| 4 | rs3775948 | 9,995,182 | 3.09E-03 |  | 0.3951 |  |
| 4 | rs3756231 | 10,025,544 | 3.30E-03 |  | 0.2656 |  |
| 4 | rs11723742 | 10,111,572 | 0.0168 |  | 0.0730 |  |
| 4 | rs28441463 | 10,289,577 | 0.0120 |  | 0.1069 |  |
| 4 | rs12505410 | 89,030,841 | 1.33E-06 | rs2231142 | 0.0171 | *ABCG2* |
| 4 | rs1481012 | 89,039,082 | 8.96E-11 |  | NA |  |
| 4 | rs2231142 | 89,052,323 | 3.83E-10 |  | NA |  |
| 6 | rs11754288 | 25,776,949 | 9.58E-05 | rs1165196 | 0.8391 | *SLC17A1* |
| 6 | rs68094823 | 25,795,971 | 4.33E-06 |  | 0.2377 |  |
| 6 | rs1165196 | 25,813,150 | 1.94E-05 |  | NA |  |
| 11 | rs801733 | 65,934,549 | 0.0264 | rs4073582 | 0.9931 | *CNIH-2* |
| 11 | rs4073582 | 66,050,712 | 0.0339 |  | NA |  |
| 12 | rs11065995 | 112,100,755 | 0.0410 | rs671 | 0.1798 | *MYL2-CUX2 (ALDH2)* |
| 12 | rs11066008 | 112,140,669 | 2.94E-03 |  | 0.1703 |  |
| 12 | rs671 | 112,241,766 | 6.80E-03 |  | NA |  |

CHR, Chromosome; SNP, dbSNP rs number; BP, Position, based on hg19.

# Supplementary Table S8. Cumulative effect of seven associated variants from the gout associated loci for developing gout

| **Risk allele  Number** | **OR** | **L95** | **U95** | **P** |
| --- | --- | --- | --- | --- |
| *≤5* | *Reference* | | | |
| 6 | 0.871 | 0.266 | 2.850 | 0.8193 |
| 7 | 0.639 | 0.225 | 1.815 | 0.4000 |
| 8 | 1.310 | 0.545 | 3.152 | 0.5459 |
| 9 | 2.925 | 1.168 | 7.326 | 0.0219 |
| 10 | 4.158 | 1.632 | 10.597 | 2.82E-03 |
| 11 | 6.892 | 2.513 | 18.896 | 1.77E-04 |
| ≥12 | 16.361 | 4.826 | 55.463 | 7.22E-06 |

OR, odds ratio, for A1; L95, the lower endpoint of 95% CI; U95, the upper endpoint of 95% CI; P, p value.

We included rs1260326 (L446P) of *GCKR*, rs11722228 of *SLC2A9*, rs12505410 and rs2231142 (Q141K)of *ABCG2*, rs4073582 of *CNIH2*, rs671 (E504K) of *ALDH2* (*MYL2-CUX2*) and rs9895661 of *BCAS3* in this analysis.

# Supplementary Table S9. Cumulative effect of twelve associated variants from the SU associated loci for developing gout

| **Risk allele  Number** | **OR** | **L95** | **U95** | **P** |
| --- | --- | --- | --- | --- |
| *≤8* | *Reference* | | | |
| 9 | 1.644 | 1.076 | 2.510 | 0.0214 |
| 10 | 2.313 | 1.539 | 3.475 | 5.42E-05 |
| 11 | 3.309 | 2.103 | 5.206 | 2.28E-07 |
| 12 | 2.735 | 1.633 | 4.578 | 1.30E-04 |
| 13 | 5.020 | 2.502 | 10.070 | 5.60E-06 |
| ≥14 | 8.884 | 4.249 | 18.580 | 6.49E-09 |

OR, odds ratio, for A1; L95, the lower endpoint of 95% CI; U95, the upper endpoint of 95% CI; P, p value.

Twelve variants (rs17632159, rs6935612, rs1165196 (I269T), rs3734692, rs9321446, rs9314273, rs10821871, rs2361216, rs11172134, rs7978353, rs61168554 and rs11150190) were included in this analysis.

# Supplementary Table S10. Cumulative effect of seven associated variants from the gout and SU associated loci for developing gout

| **Risk allele  Number** | **OR** | **L95** | **U95** | **P** |
| --- | --- | --- | --- | --- |
| *≤5* | *Reference* | | | |
| 6 | 0.706 | 0.192 | 2.591 | 0.5993 |
| 7 | 1.106 | 0.343 | 3.561 | 0.8663 |
| 8 | 1.697 | 0.644 | 4.470 | 0.2849 |
| 9 | 4.325 | 1.627 | 11.497 | 3.32E-03 |
| 10 | 6.566 | 2.298 | 18.762 | 4.42E-04 |
| 11 | 13.259 | 4.092 | 42.955 | 1.64E-05 |
| ≥12 | 30.230 | 7.102 | 128.684 | 3.98E-06 |

OR, odds ratio, for A1; L95, the lower endpoint of 95% CI; U95, the upper endpoint of 95% CI; P, p value.

For the combined analysis of variants from gout and SU associated loci, we used the ≤5 bin in the gout associated loci analysis as reference, and excluded the individuals with ≤8 risk alleles in the SU associated loci analysis from the test bins (6, 7, 8, 9, 10, 11, and ≥12).

# Supplementary Methods

**Ethics.** The sample collection and the clinical information regarding the subjects were undertaken following informed consent and approval by the relevant ethics review board at the Affiliated Hospital of Qingdao University in accordance with the tenets of the Declaration of Helsinki.

**Subjects.** All of the gout patients analyzed in the study were interviewed by endocrinologists and diagnosed according to the American College of Rheumatology criteria for gout.

**Quality control of the GWAS dataset.** For array data, a dish quality control (DQC) value greater than 0.82 was set as the primary quality control step. After excluding the arrays due to DQC failure (n=94), we used Axiom Genotyping Algorithm v1 (Axiom GT1) to generate the genotype data. Additional quality control steps were conducted as follow: (1) excluded the sample with self-reported genders not match the genotyped genders; (2) excluded the samples with generated genotypes for <95%; (3) When pairs of individuals had a PI_HAT >0.25 (PLINK's identity by descent analysis), the member of the pair with the lower call rate was excluded.

A total of 1255 cases and 1848 controls were retained for further analyses. For the SNP filtering, SNPs with call rates <95%, or MAF <3% or SNPs that deviated significantly (p≤1×10^−5^) from Hardy-Weinberg equilibrium in the controls were excluded. A total of 603,697 SNPs passed the quality criteria and were used in the subsequent analyses.

**Imputation.** Ungenotyped variants of the autosomes were imputed in the post quality control GWAS data using SHAPEIT 2.0 (phasing step), and IMPUTE2 (imputation step). The haplotype information from the 1000 Genomes Project (Phase I integrated variant set across all 1,092 individuals, v2, March 2012).

**Population stratification analysis.** The population stratification was assessed using a principal component analysis (PCA)-based method implemented in the software package EIGENSTRAT, and a total of 20 principal components were generated for the correction.

**Cumulative genetic risk score analysis.** For each individual, a cumulative risk score was determined in an unweighted approach, that is, counting the number of risk alleles carried at each variant and making a summation for all the test variants. Cumulative risk scores were then grouped into several bins. Using the bin with the least risk alleles as the reference category, the OR and 95% CI for each of the other bins were assessed using logistic regression model with 20 principal components as covariates. For the analysis using variants from the gout associated loci, seven variants (rs1260326 (L446P)of *GCKR*, rs11722228 of *SLC2A9*, rs12505410 and rs2231142 (Q141K)of *ABCG2*, rs4073582 of *CNIH2*, rs671 (E504K)of *ALDH2* (*MYL2-CUX2*) and rs9895661 of *BCAS3* ) were included and eight bins (≤5, 6, 7, 8, 9, 10, 11, and ≥12) were generated. For the analysis using variants from the SU associated loci, we included 12 variants (rs17632159, rs6935612, rs1165196 (I269T), rs3734692, rs9321446, rs9314273, rs10821871, rs2361216, rs11172134, rs7978353, rs61168554 and rs11150190), and grouped the individuals into seven bins (≤8, 9, 10, 11, 12, 13 and ≥14). For the combined analysis of variants from gout and SU associated loci, we used the ≤5 bin in the gout associated loci analysis as reference, and excluded the individuals with ≤8 risk alleles in the SU associated loci analysis from the test bins (6, 7, 8, 9, 10, 11, and ≥12).

# REFERENCES

1. Matsuo H, Yamamoto K, Nakaoka H, et al. Genome-wide association study of clinically defined gout identifies multiple risk loci and its association with clinical subtypes. Annals of the rheumatic diseases 2016;**75**(4):652-9 doi: 10.1136/annrheumdis-2014-206191[published Online First: Epub Date]|.

2. Kottgen A, Albrecht E, Teumer A, et al. Genome-wide association analyses identify 18 new loci associated with serum urate concentrations. Nature genetics 2013;**45**(2):145-54 doi: 10.1038/ng.2500[published Online First: Epub Date]|.

3. Dehghan A, Kottgen A, Yang Q, et al. Association of three genetic loci with uric acid concentration and risk of gout: a genome-wide association study. Lancet 2008;**372**(9654):1953-61 doi: 10.1016/S0140-6736(08)61343-4[published Online First: Epub Date]|.

4. McArdle PF, Parsa A, Chang Y-PC, et al. Association of a common nonsynonymous variant in GLUT9 with serum uric acid levels in Old Order Amish. Arthritis and Rheumatism 2008;**58**(9):2874-81 doi: 10.1002/art.23752[published Online First: Epub Date]|.

5. Sulem P, Gudbjartsson DF, Walters GB, et al. Identification of low-frequency variants associated with gout and serum uric acid levels. Nature genetics 2011;**43**(11):1127-30 doi: 10.1038/ng.972[published Online First: Epub Date]|.

6. Kolz M, Johnson T, Sanna S, et al. Meta-analysis of 28,141 individuals identifies common variants within five new loci that influence uric acid concentrations. PLoS genetics 2009;**5**(6):e1000504 doi: 10.1371/journal.pgen.1000504[published Online First: Epub Date]|.

7. Yang B, Mo Z, Wu C, et al. A genome-wide association study identifies common variants influencing serum uric acid concentrations in a Chinese population. BMC medical genomics 2014;**7**:10 doi: 10.1186/1755-8794-7-10[published Online First: Epub Date]|.

8. Matsuo H, Takada T, Ichida K, et al. Common Defects of ABCG2, a High-Capacity Urate Exporter, Cause Gout: A Function-Based Genetic Analysis in a Japanese Population. Science Translational Medicine 2009;**1**(5) doi: 10.1126/scitranslmed.3000237[published Online First: Epub Date]|.

9. Middelberg RPS, Ferreira MAR, Henders AK, et al. Genetic variants in LPL, OASL and TOMM40/APOE-C1-C2-C4 genes are associated with multiple cardiovascular-related traits. Bmc Medical Genetics 2011;**12** doi: 10.1186/1471-2350-12-123[published Online First: Epub Date]|.

10. Yang Q, Kottgen A, Dehghan A, et al. Multiple genetic loci influence serum urate levels and their relationship with gout and cardiovascular disease risk factors. Circulation. Cardiovascular genetics 2010;**3**(6):523-30 doi: 10.1161/CIRCGENETICS.109.934455[published Online First: Epub Date]|.

11. Chiba T, Matsuo H, Kawamura Y, et al. NPT1/SLC17A1 Is a Renal Urate Exporter in Humans and Its Common Gain-of-Function Variant Decreases the Risk of Renal Underexcretion Gout. Arthritis & Rheumatology 2015;**67**(1):281-87 doi: 10.1002/art.38884[published Online First: Epub Date]|.

12. Tin A, Woodward OM, Kao WH, et al. Genome-wide association study for serum urate concentrations and gout among African Americans identifies genomic risk loci and a novel URAT1 loss-of-function allele. Human molecular genetics 2011;**20**(20):4056-68 doi: 10.1093/hmg/ddr307[published Online First: Epub Date]|.

13. Sakiyama M, Matsuo H, Nakaoka H, et al. Identification of rs671, a common variant of ALDH2, as a gout susceptibility locus. Scientific Reports 2016;**6** doi: 10.1038/srep25360[published Online First: Epub Date]|.

14. Wallace SL, Robinson H, Masi AT, Decker JL, McCarty DJ, Yu TF. Preliminary criteria for the classification of the acute arthritis of primary gout. Arthritis and rheumatism 1977;**20**(3):895-900 doi: 10.1002/art.1780200320[published Online First: Epub Date]|.

15. Delaneau O, Marchini J, Zagury J-F. A linear complexity phasing method for thousands of genomes. Nature methods 2012;**9**(2):179-81

16. Howie BN, Donnelly P, Marchini J. A flexible and accurate genotype imputation method for the next generation of genome-wide association studies. PLoS genetics 2009;**5**(6):e1000529

17. Price AL, Patterson NJ, Plenge RM, Weinblatt ME, Shadick NA, Reich D. Principal components analysis corrects for stratification in genome-wide association studies. Nature genetics 2006;**38**(8):904-09
